# Higher-Order Logical Reasoning in Preschool Children: Evidence from Intonation and Quantifier Scope

**DOI:** 10.12688/openreseurope.18312.1

**Published:** 2025-02-03

**Authors:** Uli Sauerland, Ayaka Sugawara, Kazuko Yatsushiro

**Affiliations:** 1 Leibniz-Centre General Linguistics, Berlin, Germany; 2 Waseda University, Tokyo, Japan

**Keywords:** children, language, reasoning, intonation, scope, alternatives, negation, quantification, German

## Abstract

**Background:**

Logical reasoning in young children is difficult to ascertain experimentally even for single propositional operators. We present a novel argument that four- and five-year old children are capable of reasoning with complex representations containing multiple logical operators.

**Methods:**

The argument is based on an interaction between sentence interpretation and intonation. This interaction depends on the computation of logical inferences between the sentence uttered and possible alternative utterances containing proportional generalized quantifiers, and how adults arrive at different interpretations is well understood. The account that explains the interaction predicts that a specific intonation will disambiguate scopal interpretation in sentences with a negation and a universal quantifier, but not in sentences involving two quantifiers.

**Results:**

We show that preschool children speaking German are sensitive to the interaction between logical scope of expressions and intonation in the same way as adult speakers.

**Conclusion:**

This result entails that preschool children can carry out logical reasoning within a higher order logic.

## Plain language summary

We tend to associate logical reasoning with activities such as proving mathematical theorems or developing scientific theories. But logical reasoning can also often be observed in a more indirect way. Here we built on the widely accepted linguistic result that adults crucially use higher order logical reasoning to disambiguate sentences like “All politicians are not corrupt” on the basis of intonation. We introduce a task where participants interpret similar sentences in German, where we also vary the intonation. We show that preschool children can integrate the intonation with the quantifier interpretation similarly to how adults do it. Because we also test further control conditions, we conclude that preschool children must be employing higher order logical reasoning in the same way as adults do. Our result is the first to provide evidence for higher order logical reasoning in five year old children.

## Introduction

The ability to think logically is a core part of our identity as *homo sapiens* and a central interest for understanding human nature. Its origins and development in children are the topic of much research. Recent work in cognitive science has provided evidence that prelinguistic infants and even members of other species can successfully carry out inferences that indicate an understanding of propositional logic, specifically negation and the disjunctive syllogism ( Cesana-Arlotti *et al.*, 2018; Cesana-Arlotti *et al.*, 2020; Mody & Carey, 2016). Similar work has revealed some understanding of propositional logic even in other species including all the great apes ( Call, 2004), baboons ( Dautriche *et al.*, 2022), and grey parrots ( Pepperberg *et al.*, 2019). There are many logical systems of different orders of formal expressivity with propositional logic being only the most basic ( Frege, 1879). An important research question is how, if at all, the order of logical systems is reflected in cognitive development or evolution. Current work leaves it open at what age reasoning requiring first and higher order logic is acquired in children, however.

### The orders of logic

Over millennia, philosophers and mathematicians have developed a variety of formal calculi that can capture logical inference relations in different domains ( Kneale & Kneale, 1962). The two most widely known systems are propositional (or Boolean) and first order (or predicate) logic. Propositional logic has primitives that capture propositional constants and the meanings of conjunction ( *‘and’*, *∧*), disjunction ( *‘or’*, *∨*), and negation ( *‘not’*, *¬*), which operate on one or two propositions. Recent findings on early logical cognition exclusively concerned propositional logic, primarily disjunction, negation ( Cesana-Arlotti *et al.*, 2018; Feiman *et al.*, 2022) ^1^ and modality ( Leahy & Carey, 2020). First order logic includes at least the additional primitives *∀* for universal (‘for all’) quantification, *∃* for existential (‘exists’) quantification, as well as *=* for identity (‘equals’) and bound variables. First order logic is a strictly more expressive system than propositional logic, but it still cannot express concepts such as infinity (‘Infinitely many *x* are *P*’) or a proportion (‘More than half of *x* are *P*’). Only a higher order logic can express these concepts ( Boolos, 1984; Westerståhl, 2019). Our focus is on higher order logical reasoning that involves proportional quantification that concepts expressed by *most* or *more than half* by adults. Though children do not know these words, we present novel evidence that children must

### Quantification in language

One way quantification can be expressed in English (and many other languages) is via quantificational determiners such as *all*, *some*, *none*, *many*, and *most* ( Bach *et al.*, 1995). At least the quantifiers of first order logic seem to be expressible in all natural languages including newly emerged languages ( Kocab *et al.*, 2022). The meaning of quantificational determiners has been analyzed primarily not within first order logic, but by the addition of generalized quantifiers to first order logic ( Barwise & Cooper, 1981; Montague, 1970). While a quantifier *Q _FO_* in first order logic combines with a single property *σ* as in *Q _FO_x . σ*( *x*), a generalized quantifier *Q _GQ_* combines with two properties *ρ* and *σ* as in *Q _G_*_*Q*_( *ρ*)( *σ*) ( Lindström, 1966; Mostowski, 1957). Many generalized quantifiers can, however, also be expressed in first order logic and vice versa ( Peters & Westerståhl, 2006). For example, the generalized universal quantifiers *∀ _G_*_*Q*_, *∃ _GQ_* and two _*GQ*_can be captured within predicate logic itself:

[1] *∀ _GQ_* ( *ρ*)( *σ*)is true iff. *∀x*( *ρ*( *x*) *→* *σ*( *x*))

[2] *∃ _GQ_* ( *ρ*)( *σ*)is true iff. *∃x*( *ρ*( *x*) *∧* *σ*( *x*))

[3] two _*GQ*_( *ρ*)( *σ*)is true iff. *∃x ∃y* ( *x* ≠ *y *∧* ρ*( *x*) **∧* σ*( *x*) **∧* ρ*( *y*) **∧* σ*( *y*))

Because the first order quantifiers *∀* and *∃* can both be expressed by generalized quantifiers, first order logic can be subsumed by a logic with generalized quantifiers. But generalized quantifiers are more expressive than first order quantifiers: proportional relations can only be captured with generalized quantifiers. For example, the generalized quantifier *most _GQ_* that expresses ‘more than half of *ρ* are also *σ*’ cannot be expressed in first order logic ( Kolaitis & Väänänen, 1995; Resher, 1962):

[4] *M _GQ_* ( *ρ*)( *σ*) is true iff. *|{x | ρ*( *x*) *∧* *σ*( *x*) *}| > |{x | ρ*( *x*) *∧* *¬σ*( *x*) *}|*

Human adult logical competence as revealed by language must not only exceed first order logic, but requires higher order logic, because proportional quantifiers exceed the expressivity of first order logic. What about children though?

### Evidence from children’s quantifier word comprehension

At what age do children master proportional quantification? For first-order quantifier words such as English *a*, *some* and *all*, several studies show that children understand their core logical meaning before age 5, though independent factors may mask this understanding in more complex sentences ( Aravind *et al.*, 2017; Crain & Thornton, 2000; Foppolo *et al.*, 2012; Huang & Snedeker, 2009). Results from some studies that directly compare *most* with existential and universals suggest that first-order quantifier words are acquired faster than higher-order *most* ( Sullivan *et al.*, 2018)

As for proportional quantifiers, existing results seem to indicate that higher order logical relations are not yet fully acquired at the age of 5 years old ( Sullivan *et al.*, 2018). The evidence underlying this conclusion is comprised of a series of results on children’s understanding of the quantifier *most* and equivalent expressions in other languages ( Barner *et al.*, 2009; Katsos *et al.*, 2016; Papafragou & Schwarz, 2006). While earlier studies showed mixed results, Sullivan *et al.* (2018) report three studies using the give-a-set-task ( Wynn, 1990), where children are asked for a set of items to give a certain quantity to the experimenter. They report that English-speaking children up to age 6 show no evidence of understanding sentences with the quantifier *most* differently from sentences containing the non-word *blick* used as a quantifier, while adults do: 4-year olds reached at least 78% adult performance on the singular existential *a* and the universal *all*. While Sulllivan *et al*.’s study is based only on English, Katsos *et al.* (2016) present evidence that 5 to 6-year old children speaking 31 different languages exhibit consistently higher performance judging truth and falsity of universally and existentially quantified statements than for statements with the proportional quantifier equivalent to *most* using a different method (namely, the truth-value judgement task; Crain & Thornton, 2000). Only 60.8% of the 768 children (average age 66 month) correctly rejected a statement such as ‘Most of the apples are in the boxes’ if only two out of five are in the boxes while the same children’s accuracy on the singular existential *a* and the universal *all* is above 80% across languages.

Children’s failure with *most* and its translations into other languages, however, may also reflect other factors such as the internal complexity of *most* and its counterparts in other languages ( Hackl, 2009). If this is the case, it would then not necessarily provide an accurate measure of children’s facility with higher-order logic. We propose a new method based on the implicit reasoning underlying an interaction of scope ambiguity and intonation to provide evidence of higher order logical reasoning in four- to five-year-old children. We first present the linguistic account of this interaction in adults.

### Scope ambiguity

While logical formulas unequivocally indicate operator scope, natural language sentences containing multiple logical words can be ambiguous with respect to their scope. We discuss two examples of this type that play a role in the remainder of the paper: *all-not* sentences and *one-all* sentences. Example [ 5] illustrates *all-not* sentences which involve a universal quantifier *all* and negation *not*. The ambiguity of sentence [ 5] is captured by the two logical formula [ 6] and [ 7]. In the so-called *surface scope* interpretation in [ 6], the scope of the universal quantifier and negation corresponds to the linear order of *all* and *not* in the sentence [ 5], while the scope of universal quantifier and negation is the inverse of the linear order of *all* and *not* in the *inverse scope* interpretation in [ 7].

[5] *All T-shirts are not dried.*

[6] surface scope: *∀ _GQ_* (T-shirt)( *λx . ¬*dried( *x*))

[7] inverse scope: *¬∀ _GQ_* (T-shirt)( *λx .* dried( *x*))

**Figure 1.**
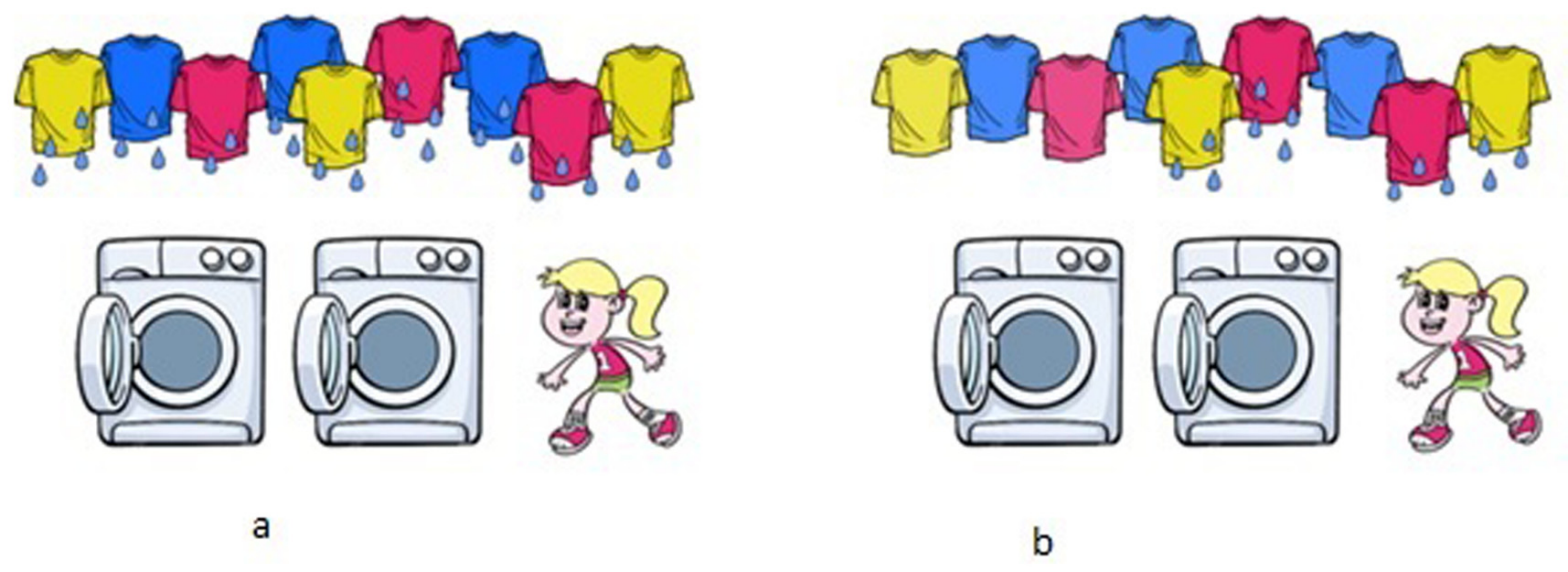
Scenarios to test the scope ambiguity of the *all-not* sentence [ 5] ‘All T-shirts are not dried.’ Reading [ 6] is true in scenario **a**. and false in scenario **b**., while reading [ 7] is true in scenario **b**.

Figure 1 shows two scenarios that distinguish between the two interpretations of [ 5]. The surface scope interpretation [ 6] is true for scenario 1-a and false for 1-b. The inverse scope interpretation [ 7], on the other hand, is true for scenario 1-b, while it is also logically true for scenario 1-a. ^2^

An example of a *one-all* sentence is shown in [ 8]. For this sentence, the relative scope of the existential *one* and the universal *all* can either be surface scope in the logical configuration of *∃* having scope over *∀* in [ 9] or inverse scope in [ 10].

[8] *One boy has picked all the flowers*.

[9] *surface scope*: *∃ _GQ_* (boy)( *λx . ∀ _GQ_* (flower)( *λy .* picked( *x,y*)))

[10] *inverse scope*: *∀ _GQ_* (flower)( *λy . ∃ _GQ_* (boy)( *λx .* picked( *x,y*)))

**Figure 2.**
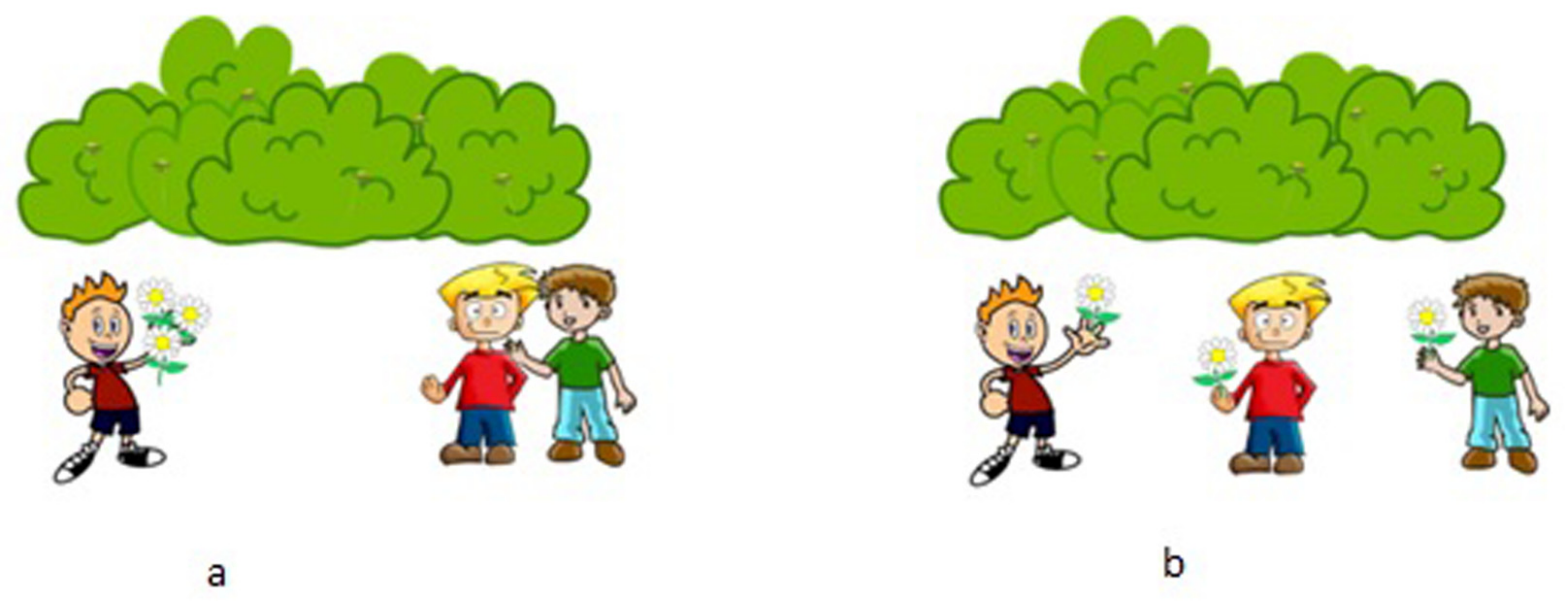
Scenarios to test the scopal ambiguity of the *one-all* sentence [ 8] ‘One boy picked all the flowers.’ Reading [ 9] is true in scenario **a**, but false in scenario **b**. Only reading [ 10] is true in the scenario **b**.

Figures 2a and 2b correspond to the two interpretations of [ 8]. The surface scope interpretation in [ 9] is true in Figure 2a but false in Figure 2b, while the inverse scope interpretation in [ 10] is logically true in both scenarios.

Important factors that constrain scope ambiguity in general are language ( Frey, 1993; Huang, 1982), sentence structure ( Rodman, 1976; Wurmbrand, 2018) and logical relations among operators ( Fox, 2000), but in the present paper, we only discuss intonation.

### Disambiguation by contrastive topic intonation

**Figure 3.**
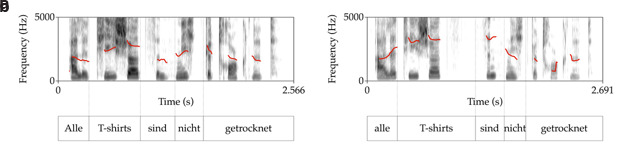
Spectrograms with a F0 pitch track (red line) of **a**. the neutral intonation contour and **b**. the contrastive topic intonation contour of the German translations of [ 5] as used in the experimental study reported. The pitch track in **b**. shows the tonal rise on *alle* (all) and a fall on *nicht (not)*. Figure 3 **a**. all T-shirts are not dried. Figure 3 **b**. all _T_ T-shirts are not _F_ dried.

While an *all-not* sentence like [ 5] in English is scopally ambiguous when uttered with neutral intonation, it becomes unambiguous, allowing for only the inverse scope interpretation in [ 7], when it is uttered with a specific intonation contour called the *B-accent* ( Jackendoff, 1972; Jespersen, 1933). A similar phenomenon is observed in German ( Jacobs, 1984), the language we investigated experimentally. In German, disambiguation in *all-not* sentences is caused by an intonation contour called the *hat intonation*. The German neutral and hat intonation contours are shown in the spectrograms in Figure 3.

The term *Contrastive Topic Intonation* refers to intonation contours like the English B-accent and the German hat contour independent of their language-specific phonetic properties ( Féry, 1993). Contrastive topic intonation in the languages where it has been studied is localized on two words (or phrases) in a sentence. We adopt the notation of underlining both accented words and furthermore marking the first with subscript T and the second with subscript F as shown in Figure 3b. Büring ( 1997, 2003) establishes a widely-accepted explanation of the scope disambiguation of *all-not* sentences by contrastive topic intonation. The explanation is based on the observation that the hat contour also occurs in answers to questions that contain multiple interrogative particles ( Bolinger, 1965): For example, in answer to the question *Who drank what?*, the underlined words would receive the contrastive topic accents — Chris _T _drank coffee _F _and Kai _T _drank tea _F_.

We summarize Büring’s proposal in the following condition:

[11] A statement *S* with contrastive topic intonation is licit if and only if there is a question *Q* targeting the two elements marked by the contrastive topic intonation of *S* such that *S* partially, but not completely answers *Q*.

The scope disambiguation in the *not-all* sentence in [ 5] follows from condition in [ 11] via a sequence of inferences. The following question in [ 12] targets negation and the universal in the sense of [ 11]:

[12] *Q*_1_ = For what *n* are at least *n*% of the T-shirts dried or not dried? 

The question *Q*_1 _is completely answered by interpretation in [ 6] for sentence [ 5], however, because if All / 100% of the T-shirts are such that they are not dried, it is also the case that for any number *x <* 100, at least *x*% of the T-shirts must be not dried. Therefore condition in [ 11] would not be satisfied if the speaker uttering [ 5] intended the surface scope interpretation in [ 6] (All the t-shirts are such that they are dry). Interpretation [ 7], however, answers *Q*_1 _only partially, not completely: That it is not the case that 100% of the T-shirts are dried provides a partial answer to *Q*_1_, but not a complete one because it still remains open whether or not, for example, at least 90% of the T-shirts are dried. Therefore condition in [ 11] is satisfied if the inverse scope interpretation in [ 7] is intended. Hearers who apply this reasoning are then correctly predicted to infer that the intended interpretation for sentence [ 5] with contrastive topic intonation must be [ 7]. Importantly the correct disambiguation of *all-not* sentences is only predicted if listeners go through a chain of logical reasoning with proportional quantifiers—i.e. the account relies on higher order quantificational reasoning. ^3^

### No disambiguation in *one-all* sentences

Statements like [ 8] are not disambiguated by contrastive topic intonation ( Krifka, 1998) as the requirement in [ 11] correctly predicts. Specifically, a question corresponding to [ 8] in the sense of [ 11] is *Q*_2_:

[13] *Q*_2 _= For what *n* and for what *m* did *n* of the boys pick *m*% of the flowers? 

But both interpretations in [ 8] answer the question *Q*_2 in _in [ 13] only partially: (i) the surface scope interpretation in [ 9] leaves open whether any of the boys picked multiple flowers, and (ii) the inverse scope interpretation in [ 10] leaves open whether multiple boys picked some or all of the flowers. Condition in [ 11], therefore, predicts correctly that the contrastive topic intonation does not disambiguate *one-all* sentences. The difference in the availability of disambiguation between *all-not* sentences and *one-all* sentences provides crucial evidence that German adults apply higher order logical reasoning as required by condition [ 11] to interpret contrastive focus.

### A novel test for higher order logic

Our novel test for the higher order logical reasoning in children applies therefore scope disambiguation by intonation. If children exhibit the same pattern of disambiguation for *all-not* and *one-all* sentences as adults do, this would argue that children can apply the same higher order reasoning as adults do. Lohiniva & Panizza (2016) and Sugawara *et al.* (2018) investigate scope disambiguation by intonation by German and English speaking children (4;4-6;10) respectively. However both studies test only *all-not* sentences like [ 5], not *one-all*, leaving the picture incomplete. Specifically, recent research lead us to be skeptical as to whether children could successfully apply condition [ 11] for the following three reasons. First, prior research by Musolino (1998) on scope ambiguity in children has endorsed the view that children can only access interpretations where the linear order in the sentence corresponds to the scopal order of logical elements ( Gualmini *et al.*, 2008; Viau *et al.*, 2010, and others). Secondly, other research has found that children are not as sensitive to intonation as adults in various cases of disambiguation: Choi & Mazuka (2003) report on 5- to 6-year-old Korean children’s phrasal segmentation disambiguation, Snedeker & Trueswell (2001) study 4- to 6-year-old American children’s disambiguation of two potential syntactic attachment sites, and ( Crain *et al.* (1994), Costa & Szendrői (2006), and Sekerina & Trueswell (2012) considered focus intonation in association with a focus particle or as contrastive topic (cf. Szendrői *et al.*, 2018; Zhou *et al.*, 2012). Finally, some prior research argues that children could not access alternatives to a sentence they hear ( Barner *et al.*, 2011; Chierchia *et al.*, 2001), but condition [ 11] requires access to alternative with a different proportional quantifier. In sum, it seems initially implausible that children should be capable of relying on condition [ 11] to interpret contrastive topic intonation. Instead it seems plausible that they might rely on a less complex alternative strategy such as the following:

[14] Contrastive topic intonation indicates that an inverse scope intonation is intended.

To distinguish whether children rely for scope disambiguation on condition [ 11] or on [ 14], *some-all* sentences provide a crucial control. For the *all-not* example in [ 5], strategy [ 14] makes the same predictions as [ 11]: children should disambiguate [ 6] when it is produced with a contrastive topic intonation as [ 5]. But the strategy [ 14] would predict that contrastive topic intonation should also disambiguate *one-all* sentences like 8 towards the inverse scope interpretation. As we noted above, adults do not disambiguate [ 8] even with contrastive topic intonation. This lack of disambiguation shows that the adult behavior could not be explained by [ 14]. We therefore compared the disambiguation effect of contrastive topic in *all-not* and in *one-all* sentence with child and adult participants– the crucial comparison to test for higher order logical reasoning.

## Methods

### Experimental manipulations

**Figure 4.**
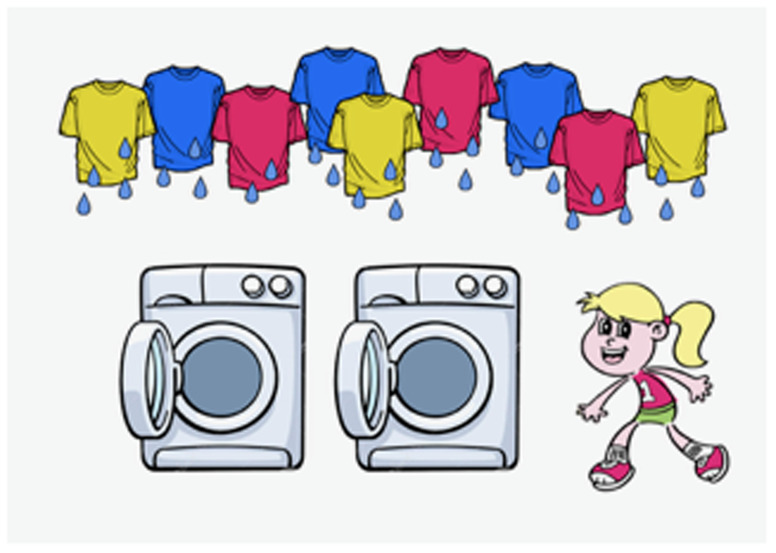
Introductory picture for the example item 15.

The experiment was based on the design by Sugawara *et al.* (2018), using a picture selection paradigm. The experiment was conducted with each participant individually using a presentation program (Keynote) on a tablet (iPad, Apple). Audio stimuli were pre-recorded by a trained female native German speaker and played from the loudspeaker of the tablet used for the experiment. Each item consisted of two slides: the first to set up the relevant context, and the second showing four possible outcomes. The experimental procedure was designed to portray a conversation between a protagonist on the slide and the experimenter. The conversation was designed to set up the right context/question under discussion to which the experimental sentence that is uttered by the protagonist is at least a partial answer ( Büring, 1997). An example is shown in [ 15] (see supplementary information for all items). Participants see a picture of a protagonist with some objects (e.g., a girl with many wet T-shirts in the background; Figure 4), and hear the context from her perspective.

[15] *Ich habe viel zum Waschen heute. Ich bin gerade damit fertig geworden die*

*T-Shirts zu waschen, aber sie sind immer noch nass.*

      ‘I have a lot of laundry today. I just finished washing the T-shirts but they’re still wet.’

**Figure 5.**
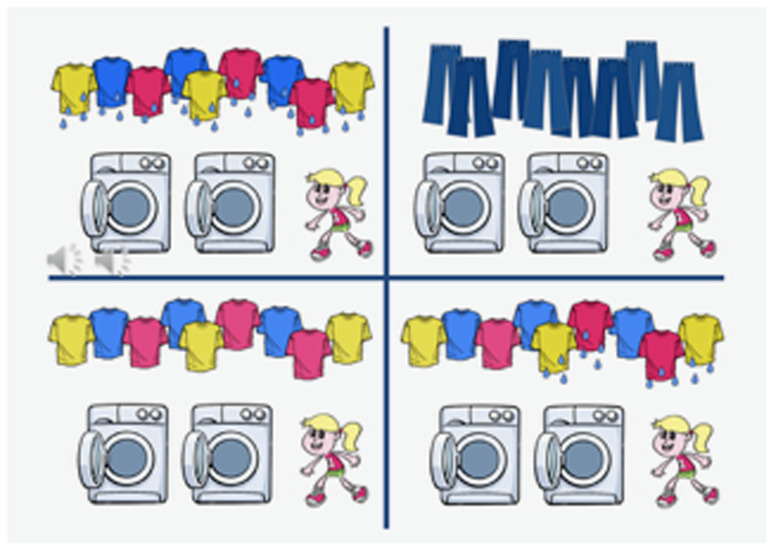
Sample picture presented with sentence [ 5].

The second slide (in Figure 5) was partitioned into four parts, each part showing pictures as follows:

(1) a. surface scope picture (all *≫* not)
b. inverse scope picture (not *≫* all)
c. positive picture (that correspond to the test sentence without the negation; e.g. all t-shirts are dry: False)
d. Irrelevant situation (e.g. all jeans are dry: Presupposition Failure)

After the experimenter asks the protagonist a question ( *Sind alle T-Shirts getrocknet?* ‘Have all the T-shirts dried?’), the protagonist responds to the question, using the experimental sentence with either the neutral or the hat intonation contour: *Alle T-shirts sind nicht getrocknet.* ‘All the T-shirts are not dry.’ The participant is then asked to choose one of the pictures that matched the statement by the protagonist.

There were two familiarization items to introduce the task to the participant. There were eight test items with *all* and negation, six test items with two quantifiers, and six fillers, resulting in 22 items in total. The order of the test and filler items was pseudo-randomized. The position of surface scope and inverse scope pictures were balanced among the four corners in the 14 test items.

The responses were recorded manually on a paper response sheet, indicating which picture the participant chose for each item. We assigned a number for each section of the monitor (1 for the top left to 4 for bottom right) and the experimenter entered the number assigned to the section on the response sheet, to avoid confusion. After the experiment, the experimenter entered them on a spreadsheet. The full experimental materials are openly accessible as part of ( Yatsushiro *et al.*, 2025 [Dataset]).

### Units of delivery and analysis

We created two versions of the experiment. For each version, only one of the intonation contours was used throughout the experiment in order to avoid one type of intonation contour affecting another item with the same syntactic configuration but produced with a different intonation contour.

### Informed consent

In accordance with the Declaration of Helsinki and other regulations, we obtained written informed consent from participants. The data were originally collected in the project “Working memory and structural knowledge” (PI K. Yatsushiro). The ethics committee of the German linguistics society (DGfS) revied and approved the project’s procedures for informed consent, participant rights and experiment design (approval date: 23.09.2014). The data we report on were collected in the years 2017 to 2019. For adult participants, written informed consent was obtained before participation. For child participants, we first obtained written informed consent from their legal guardians by sending home a consent form through their daycare. In addition, child participants were informed verbally in a child-appropriate manner of their rights as participants and asked to consent to participate.

## Results

### Participants

We tested 36 monolingual German speaking children (3;7–5;11, *M*=4;10 mo), and 20 adult speakers as the control group. Participants were randomly assigned by coin-flip to two lists (the neutral and the hat intonation contours) and therefore the two group sizes were not identical. 20 children (4;0–5;11, *M*=4;10) and 11 adults were tested with the neutral intonation contour and 16 children (3;7-5;4, *M*=4;8) and 9 adults were tested with the hat intonation contour. The children selected by chance for the critical task with hat intonation had a numerically younger mean age but the difference in mean age between the two groups was not statistically significant by a *t*-test ( *p* = *.*05114). All participants completed the experiment. The full results are openly accessible as part of ( Yatsushiro *et al.*, 2025 [Dataset]).

## Discussion

The figures in 6 show the proportion of inverse scope assignment from 36 child and 20 adult participants. The two left plots in both panels show the results from adult speakers for either contrastive topic (pink) or neutral (blue) intonation, while the two right plots show the results from children. Each dots represents a single speaker’s mean on six items.

We separated data from child and adults participants, and fitted a generalized linear mixed models (logit) with the response type (inverse scope picture vs. surface scope picture) as the dependent variable and the intonation pattern as the fixed effect. We found the effect of intonation pattern with the *all-not* sentences for both adults (z-value: *−*4 *.*795, *p < .*01) and children (z-value: *−*3 *.*645, *p < .*01). This effect is predicted by condition [ 11] but not by the alternative [ 14]. With the *one-all* sentences we did not find any effect of the intonation with adults (z-value: 0, *p* = 1) as expected by both [ 11] and by the alternative [ 14]. There was a significant difference with children (z-value: 2 *.*307, *p < .*05). The direction of the difference, however, was in the opposite direction of what the alternative [ 14] hypothesis predicts. Namely we found more inverse scope interpretations with neutral intonation than with the hat intonation contour. The analysis scripts are openly accessible as part of ( Yatsushiro *et al.*, 2025 [Dataset]).

**Figure 6.**
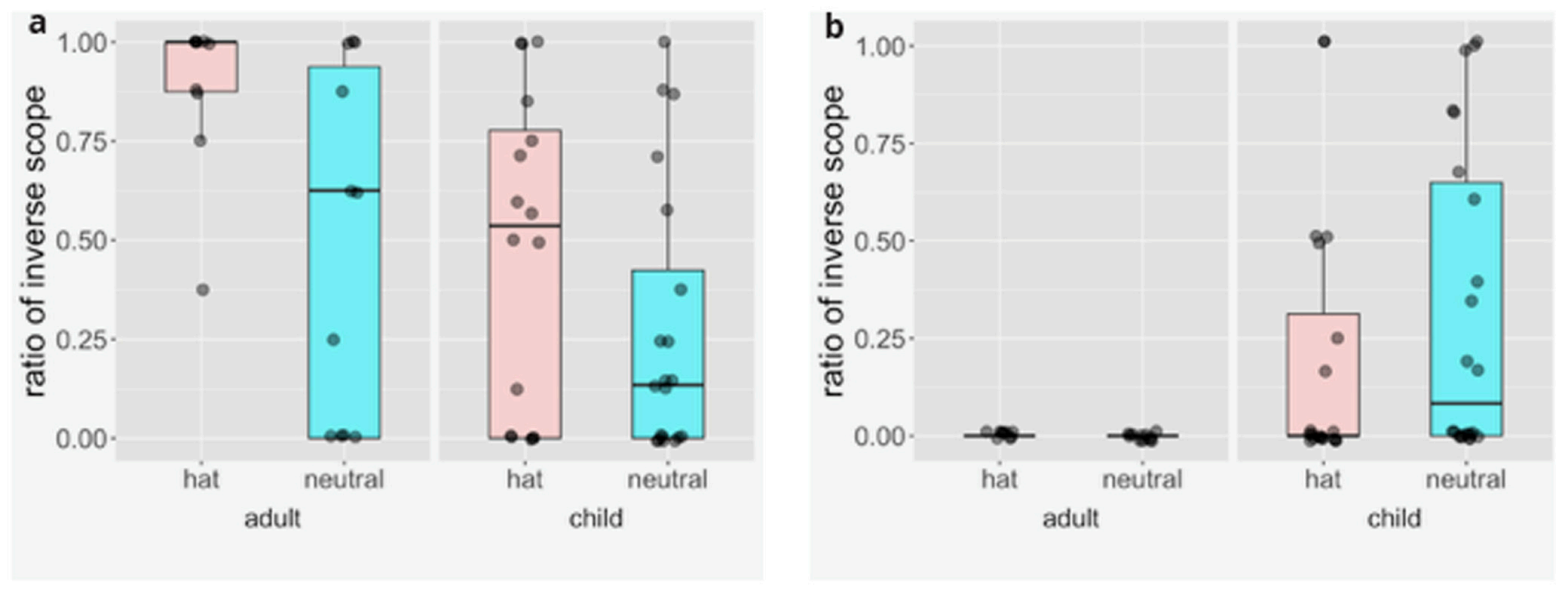
Ratio of inverse scope picture choices relative to all all choices of a surface or inverse scope picture for **a**. *all-not* sentences like [ 5] and **b**. *one-all* sentences like [ 8]. The two left plots in both panels show the results from adult speakers for either contrastive topic (pink) or neutral (blue) intonation while the two right plots show the results from children. Each dot represents a single speaker’s mean on six items.

Interpretation: We found that both adults and children are sensitive to contrastive topic intonation in the interpretation of *all-not* sentences, and also both adults and children show no sensitivity to the contrastive topic intonation in the interpretation of *one-all* sentences, indicating that both adults and children rely on condition 11 for interpreting contrastive topic and successfully follow through the higher-order logical reasoning this involves.

Children behaved slightly differently from adults especially on the control condition, but not in a way affecting the conclusion. Namely, adults showed no evidence for ambiguity of *one-all* sentences, and furthermore, they always chose the picture representing the surface scope interpretation in [ 9]. This result, we think, reflects a preference for an interpretation where scope relations reflect the linear order of quantifier words ( Fox, 2000; Frey, 1993; Jackendoff, 1972). We find a higher rate of acceptance of the alternative picture by children. Children seem not to be sensitive to this preference to the same extent as adults are, though they too prefer the surface scope interpretation. For Japanese, a similar difference between adults and children has been reported and attributed to the suggestion that children can more freely assign quantifier scope than adults can ( Yamakoshi, 2013).

Using quantifier scope disambiguation by contrastive topic intonation, we found that even four- to five-year old children are capable of reasoning with proportional quantifiers as they apply condition 11. As far as we are aware, our result is the first to reveal first or higher order logical reasoning at such a young age. Specifically the method we used shows knowledge of proportional quantification at a younger age than direct tests of the meaning of *most* do.

Our results show that the order of logics by expressivity provides a useful framework for the investigation of reasoning skills as Pepperberg (2020) also argues from a cross-species perspective. The first vs. higher order logic distinction has also been used to account for differences between singular ( *every, each*) and plural ( *all*) universal quantifiers ( Knowlton *et al.*, 2022). The order of logics, however, correlates strongly with other potential factors underlying reasoning performance such as computational complexity and numerical ability. For example, *most* is not computable by finite automata without counters ( van Benthem, 1986). At the same time, 11 months olds show evidence of approximate numerical representations of proportions ( Denison & Xu, 2014) and of expressions of comparison at age 3 ( Hohaus *et al.*, 2014). A more fine-grained formal understanding of logical-arithmetical systems ( van Benthem & Icard, 2023) should aid future cognitive work on logical reasoning.

## Ethics and consent

IRB: Ethics committee of the German linguistics society (DGfS), Approval date: September 23, 2014, Approval number: not applicable. The ethical approval letter is provided as part of ( Yatsushiro *et al.*, 2025 [Dataset]) at https://doi.org/10.17605/OSF.IO/43GHX.

## Data Availability

Open Science Foundation (OSF): Intonation and Logic. https://doi.org/10.17605/OSF.IO/43GHX. This project contains the following underlying data: intonation_presentations.zip, zip-archive of experimental stimuli for both treatment groups in original Apple Keynote format and Microsoft Powerpoint format, soundfiles.zip, zip-archive of all audiofiles used in the experimental stimuli in wav-format, int-data.csv, anonymized raw data collected; Ethikvotum.pdf, IRB letter indicating ethical approval; intonation_final.Rmd, analysis and visualization scripts used; and intonation_final.html, full result report of the analysis and visualization scripts. Data are available under the terms of the Creative Commons Attribution 4.0 International license (CC-BY 4.0, https://creativecommons.org/licenses/by/4.0/) as part of ( Yatsushiro *et al.*, 2025 [Dataset]). There are no data available other than those in ( Yatsushiro *et al.*, 2025 [Dataset]). This publication follows the Journal Article Reporting Standards for Quantitative Research in Psychology ( Appelbaum *et al.*, 2018, https://doi.org/10.1037/amp0000191, license: copyright 2018 the American Psychological Association).
