# Supplementary material for: Higher-Order Logical Reasoning in Preschool Children: Evidence from Intonation and Quantifier Scope
Source: Open Res Eur. 2025 Feb 3;5:34. [Version 1] doi: 10.12688/openreseurope.18312.1 (PMC12086508; doi:10.12688/openreseurope.18312.1)
Supplement: Peer review history

# Reviewer report: Reviewer response for version 1

**DOI:** 10.21956/openreseurope.19789.r52814

**Published:** 2025-05-16

**Authors:** Myrto Grigoroglou

**Affiliations:** 1 University of Toronto, Toronto, Ontario, Canada

The study investigates whether preschool children can compute higher-order logical interpretations involving quantifier scope (e.g., interpreting sentences like *“*All T-shirts are not dried *"* and “One boy picked all the flowers” either with surface-level or inverse scope). The authors use intonation cues (prosody) as a key variable to test whether children access surface-scope and inverse-scope readings similarly to adults. Results showed that adults interpreted *all…not* sentences with inverse scope, with both neutral and contrastive intonation, while they interpreted *one…all* sentences with surface scope. Children, similarly to adults, also tended to interpret *all…not* sentences with inverse scope but more often with contrastive intonation. Interestingly, children, unlike adults, they also had some inverse-scope readings for *one…all* sentences.

 The paper has merit both theoretically and empirically. The study has a strong theoretical framework; one of its greater strengths is the combination of formal semantic theory with psycholinguistic methodologies to answer questions about the availability of higher order logic in children by focusing on ambiguities in quantifier scope. The paper explains nicely the contribution of contrastive topic intonation in quantifier scope disambiguation by connecting formal semantics and phonology. The study is also robust experimentally; it employs a picture-selection task with carefully designed stimuli, which can account for the two types of scope interpretation of quantifiers, and are appropriate for testing child participants. The intonation manipulation is clever and relatively understudied in child language research. The statistical analyses are appropriate for the data and the authors have made all the stimuli, data and analysis code available for review. I only have a few suggestions to improve clarity in an already well-written paper.

**Introduction**

 The introduction of the paper was very well-written and theoretically rich. Overall, a very enjoyable read. Some parts could use some further explanation as I mention below.

 p. 3-4 The authors describe a study by Sullivan et al. (2018) and they mention that this study finds no evidence that children treat the quantifier most as different from the novel quantifier “blick”. However, it is not clear what point is tried to be made here. Some additional information is required.

 The section “A novel test for higher order logic” felt very condensed and could benefit from significant unpacking. Specifically, the studies mentioned at the bottom of page 6 are telegraphically described and the reader does not understand what argument each study is trying to support. A longer description of the findings of each study and more concrete examples would help the reader understand the prior literature better. Also, there seem to be some notable omissions of recent work that has looked at the role of intonation in the generation of alternatives which is critical to the present work. Some discussion of this research in the introduction and the discussion (to draw some more generalizable conclusions for the role of intonation in meaning) would enrich the theoretical impact of the study. Some references are provided below (but a more comprehensive literature review is needed here). Refer 1 to 3.

 Although, a persistent reader could reconstruct the predictions for each type of sentence and each type of intonation pattern across sections, the paper could benefit greatly from a reiteration of the predictions for the neutral and contrasting contours, for each type of quantifier sentence across age groups. Relatedly on the second column of page 6, the authors mention that “some-all sentences provide a crucial control” but do not explain why this control is crucial and what the predictions for these types of sentences are. Some explanation here would be very helpful.

**Results and Discussion**

 The results and discussion were not very easy to follow. It is certainly not ideal that the detailed statistical tests of the study can only be found in supplemental materials. Also, due to the lack of explicit predictions for the neutral intonation, it is difficult to assess what the findings actually show. Adding explicit predictions earlier would make the interpretation of the findings more straightforward.

 Overall, the discussion felt underwhelming compared to the introduction which was very detailed and theoretically rich. This section would benefit from a more analytic discussion of the findings one-by-one and their interpretation – especially when child data diverge from adult data. For example, it is not clear why intonation has a greater effect in children’s inverse scope interpretations in *all…not* sentences than adults or why children have more inverse scope preferences than adults for *one…all *sentences. Some further explanation is needed here.  Finally, the Discussion section could benefit from a more in-depth analysis of how the present findings fit with the overall findings on children’s comprehension of quantifiers and the interaction of semantics and intonation.

If any, are all the source data and materials underlying the results available?

Yes

Is the work original in terms of material and argument?

Yes

Is the work clearly and cogently presented?

Yes

Is the argument persuasive and supported by evidence?

Yes

Does the research article contribute to the cultural, historical, social understanding of the field?

Yes

Does it sufficiently engage with relevant methodologies and secondary literature on the topic?

Partly

Reviewer Expertise:

semantics, pragmatics, psycholinguistics, conceptual development

I confirm that I have read this submission and believe that I have an appropriate level of expertise to confirm that it is of an acceptable scientific standard, however I have significant reservations, as outlined above.

## References

[1] : When (not) to Look for Contrastive Alternatives: The Role of Pitch Accent Type and Additive Particles. *Lang Speech* .2019;62(4) : 10.1177/0023830918814279 751-778 10.1177/0023830918814279 30514147

[2] : Why Do Children Think Words Are Mutually Exclusive?. *Psychol Sci* .2024;35(12) : 10.1177/09567976241287732 1315-1324 10.1177/09567976241287732 39570584

[3] : Intonation and Pragmatic Enrichment: How Intonation Constrains Ad Hoc Scalar Inferences. *Lang Speech* .2017;60(2) : 10.1177/0023830917716101 200-223 10.1177/0023830917716101 28697695

# Reviewer report: Reviewer response for version 1

**DOI:** 10.21956/openreseurope.19789.r52811

**Published:** 2025-05-16

**Authors:** Daniele Panizza

**Affiliations:** 1 University of Messina, Messina, Italy

The article “Higher-Order Logical Reasoning in Preschool Children: Evidence from Intonation and Quantifier Scope” presents an experimental study investigating the interpretation of scopally ambiguous sentences involving universal quantifiers ( *all*) and negation, in 4- to 5-year-old German-speaking children and adult controls. The main results are such that children and adults display sensitivity to the so-called hat intonation (argued to trigger contrastive topic) when judging the test sentences via a picture selection task. The condition in which sentences were presented with this intonation elicited more inverse-scope interpretations (not > all) compared to the condition in which a neutral intonation was employed.

 While the results of this study are compelling, I find several points in which this article could - or rather should - be improved. I will list these points below.

1. I don’t understand why higher-order logic is needed to compute not-all interpretations. I see that the linking hypothesis is the adoption of a Büring-like contrastive topic principle so that, if children are sensitive to this principle they must be able to compute higher-order logic operations. However, the main point of computing scope ambiguity is that the order of logical operators can be reversed, such that an all > not configuration is interpreted with negation taking scope over the universal quantifier as a result of syntactic covert movement. While I’m sympathetic with the claim that preschoolers use higher-order logic to interpret natural language, evidence for this can be found in several domains, such as, e.g., the interpretation of modals and complex modifiers requiring computation on alternatives (e.g., “only”), focus, as well as the computation of scalar implicatures.
2. In relation to the previous point, I feel that the core result of the present work is that children and adults display a comparable sensitivity to intonation. Namely, in the hat contour condition, both groups produced a higher amount of not-all choices and to a similar degree. In this respect, the authors need to discuss the presence or absence of a significant interaction between intonation pattern and group of participants.
3. In general, much space is devoted to the introduction of orders of logic and children’s interpretation of quantifiers, whereas little space is given to the discussion of the inverse-scope patterns in natural languages, their interpretation in children and adults, and the principles that are argued to drive this phenomenon. For instance, economy principles have been maintained to account for the availability of inverse-scope in certain configurations but not in others (see Fox, 2000; Mayr and Spector, 2010). This topic is very important for this study in that it is not clear whether the control condition (one-all) should trigger inverse-scope readings and whether they are predicted to be as available as those generated by all-not. In this respect, the authors need to report the one-all sentences they used as controls.
4. The description of the results is extremely short. First, the authors need to report in detail the average choices provided by the participants. I could only infer the mean ratings from the graphs and I could not find the accuracy ratings, i.e., the number of choices of the distractors (the all-picture and the irrelevant one). Second, they should report the details of the statistical analysis more in detail: the structure of the logistic regression (which random factors were included in the analysis? Did they include random intercepts and slopes? Were the interactions significant?).
5. The authors claim that “Our result is the first to provide evidence for higher-order logical reasoning in five-year-old children”. I think this claim is too strong. Several studies found that preschoolers can compute a wide range of higher-order meanings such as implicatures, complex quantifiers, interactions of quantifiers and operators, modals, modal reasoning, etc. Again, the main result of this study is that preschoolers do inverse-scope (contrary to previous findings suggesting that children can only compute surface scope interpretations, i.e., the isomorphism hypothesis) and are sensitive to contrastive topic intonation similarly to adults (contrary to previous claims that children’s sensitivity to intonation is very weak).
6. There is one argument of discussion about the reported data that has been neglected, which is why adults display a strong preference for inverse-scope with hat intonation, whereas children display a strong preference for surface-scope with neutral intonation. The authors should discuss the possible causes underlying this pattern, as well as why both children and adults display intermediate ratings for the neutral (around 60% of inverse-scope responses in adults) and hat intonation (50% of inverse-scope responses in children) conditions, respectively. What could be the underlying force driving this pattern? Low computational capacity limiting scope shift? Influence of strongest-meaning hypothesis (see Crain and Thornton, 1998)? Interaction with implicatures? Interpretation preference?
7. The Büring principle stated in (11) is not universally valid. Indeed, different languages display different intonation properties. In Romance languages like Italian, for instance, a contrastive topic intonation generally indicates wide scope of negation, regardless of whether the sentence is a partial answer to the question under discussion. E.g. “Alcuni ragazzi non hanno mangiato la frutta” (= Some boys haven’t eaten the fruits) pronounced with falling intonation on “hanno” (“have”) and the last three words destressed (“mangiato la frutta”). In this case, Inverse-scope reading is a complete/strong answer to the question under discussion (no boys ate the fruits). This is not a problem for the main argument of the article (whether hat contour triggers inverse scope no matter what), but it is definitely relevant for the discussion of the results (e.g., what principle triggers inverse scope or makes it available to adults and children).
8. Finally, there is one reason why in this experiment a relatively high rate of inverse-scope readings was produced by both adults and children, compared to previous studies. That is, the experimental sentences were preceded by an all-question such as “Have all the T-shirts dried?”. This is a yes/no question that is naturally addressed by a positive (yes, they all have dried) or negative (no, it’s not true that…) answer where negation has wide scope. To answer this question with a stronger answer (“no, none of them did!”) feels like providing a more informative statement that strengthens the simplest answer (i.e., “no, they haven’t, in fact none of them has dried”). The all-question before the experimental items could have boosted the rate of inverse-scope responses in this experiment. It is not a problem per se, nor does it provide any counter-evidence to the main findings (adults and children derive inverse-scope, this is sensitive to intonation), but it should be noticed in the paper.

If any, are all the source data and materials underlying the results available?

Yes

Is the work original in terms of material and argument?

Yes

Is the work clearly and cogently presented?

Yes

Is the argument persuasive and supported by evidence?

Partly

Does the research article contribute to the cultural, historical, social understanding of the field?

Yes

Does it sufficiently engage with relevant methodologies and secondary literature on the topic?

Partly

Reviewer Expertise:

Psycholinguistics

I confirm that I have read this submission and believe that I have an appropriate level of expertise to confirm that it is of an acceptable scientific standard, however I have significant reservations, as outlined above.

## References

[1] Crain, Stephen & Rosalind Thornton. 1998. Investigations in Universal Grammar. Cambridge, MA: MIT Press.

[2] Mayr, C., & Spector, B. (2010). Not too strong! Generalizing the scope economy condition. In Proceedings of sinn und bedeutung (Vol. 14, pp. 305-321).

[3] Fox, D. (2000). Economy and semantic interpretation (Vol. 35). MIT press.

# Reviewer report: Reviewer response for version 1

**DOI:** 10.21956/openreseurope.19789.r50863

**Published:** 2025-02-08

**Authors:** Nopita Trihastutie

**Affiliations:** 1 Widya Kartika University, Surabaya, Indonesia

This study has taken part in the history of existing studies in cognitive science and made theoretical contributions to the development of cognitive science, but the authors have not explained the practical contribution or application of the research results to other scientific fields that are still related to cognitive science.

 Related to the research participants who were pre-schoolers, I think the authors can provide recommendations for the results of this research on the science of pedagogy (e.g., language pedagogy) for early childhood education, especially in terms of the development of understanding of mother tongue in early young children. Thus, this research also touches on the social and cultural aspects of cognitive science.

If any, are all the source data and materials underlying the results available?

Yes

Is the work original in terms of material and argument?

Yes

Is the work clearly and cogently presented?

Yes

Is the argument persuasive and supported by evidence?

Yes

Does the research article contribute to the cultural, historical, social understanding of the field?

Partly

Does it sufficiently engage with relevant methodologies and secondary literature on the topic?

Yes

Reviewer Expertise:

Language, Literature, Media, and Culture

I confirm that I have read this submission and believe that I have an appropriate level of expertise to confirm that it is of an acceptable scientific standard.
